# What’s New in Ocular Drug Delivery: Advances in Suprachoroidal Injection since 2023

**DOI:** 10.3390/ph17081007

**Published:** 2024-07-30

**Authors:** Kevin Y. Wu, Angel Gao, Michel Giunta, Simon D. Tran

**Affiliations:** 1 Department of Surgery, Division of Ophthalmology, University of Sherbrooke, Sherbrooke, QC J1G 2E8, Canada; yang.wu@usherbrooke.ca (K.Y.W.);; 2 Faculty of Medicine, Queen’s University, Kingston, ON K7L 3N6, Canada; 3 Faculty of Dental Medicine and Oral Health Sciences, McGill University, Montreal, QC H3A 1G1, Canada

**Keywords:** choroid, humans, injections, intraocular, retinal diseases, cost-benefit analysis, treatment outcome, quality of life, visual acuity

## Abstract

Despite significant advancements in ocular drug delivery, challenges persist in treating posterior segment diseases like macular edema (ME) and age-related macular degeneration (AMD). Suprachoroidal (SC) injections are a promising new method for targeted drug delivery to the posterior segment of the eye, providing direct access to the choroid and retina while minimizing systemic exposure and side effects. This review examines the anatomical and physiological foundations of the SC space; evaluates delivery devices such as microcatheters, hypodermic needles, and microneedles; and discusses pharmacokinetic principles. Additionally, advancements in gene delivery through SC injections are explored, emphasizing their potential to transform ocular disease management. This review also highlights clinical applications in treating macular edema, diabetic macular edema, age-related macular degeneration, choroidal melanoma, and glaucoma. Overall, SC injections are emerging as a promising novel route for administering ophthalmic treatments, with high bioavailability, reduced systemic exposure, and favorable safety profiles. Key therapeutic agents such as triamcinolone acetonide, dexamethasone, AAV-based gene therapy, and axitinib have shown promise. The field of suprachoroidal injection is progressing rapidly, and this review article, while attempting to encapsulate most of the published preclinical and clinical studies, mainly focuses on those that are published within 2023 and 2024.

## 1. Introduction

Ocular drug delivery has evolved considerably in recent years. However, treating posterior segment and retinal disease remain challenging obstacles. Intravitreal delivery of medication has revolutionized the treatment for a variety of potentially blinding retinal diseases such as age-related macular degeneration (AMD) and diabetic macular edema (DME) [1,2]. However, this modality also exposes the lens, ciliary body, and anterior segment to unintended pharmaceutic exposures and requires frequent injections for chronic conditions [1], leading to increased healthcare costs and potential side effects [2].

Suprachoroidal (SC) space injections have emerged as a promising alternative for targeted drug delivery to the posterior segment. The suprachoroidal space (SCS) is a potential space between the sclera and choroid. It can be accessed minimally invasively, facilitating the direct delivery of drugs to the choroid and retina [3]. Furthermore, the pharmaceutical agent can be compartmentalized in the SCS, away from non-target tissues, potentially reducing adverse effects (AE). Recent advancements in imaging technologies and the development of specialized delivery devices have further supported the clinical adoption of SCS injections.

Given the rapid progress in the field of suprachoroidal injection, this review does not aim to be a comprehensive overview of the topic but rather focuses on encapsulating the majority of published preclinical and clinical studies, with a particular emphasis on those released in 2023 and 2024.

## 2. Anatomy and Pathophysiology

The choroid is a vital vascular layer located between the retina and sclera that is essential for the transport of nutrients to the retina [3]. The sclera forms the protective outer layer of the eye. It is a dense, collagenous structure with varying thicknesses [3]. Scleral thickness is a key anatomical variable for SC injections because the sclera must be precisely penetrated without damaging the underlying choroidal tissues. Typically, the sclera is the thickest posterior to the limbus and thinner near muscle insertions [4,5]. Overall, studies indicate scleral thickness remains relatively consistent at the pars plana, reducing the need for individualized needle adjustments [4,5,6].

The SCS is a potential space between the sclera and choroid, which are typically close in touch due to intraocular pressure (IOP) and connecting fibers. Although adjacent, the sclera and choroid have vastly different functional and mechanical properties. The stiff collagenous sclera has a compression modulus approximately seven-fold higher than that of the flexible choroid [7]. Together, the differing mechanical properties and lack of strong physical bonds allow the SCS to expand whether through fluid injections or mechanical cannulation. The SCS also has distinct anatomical boundaries. The SCS is limited anteriorly by the scleral spur, where the sclera adheres to the ciliary body, and extends posteriorly to the optic nerve and short ciliary arteries [3].

Optical coherence tomography (OCT) imaging innovated the field of ophthalmology and facilitated in vivo choroidal imaging. OCT is a non-invasive imaging technology that utilizes light to obtain high-resolution cross-sectional images of the retina. The SCS can be visualized with swept-source OCT (SS-OCT), which allows for deeper tissue penetration, [8] and enhanced depth imaging OCT (EDI-OCT), which allows for greater depth of field [9]. The combination of both methods, EDI SS-OCT is considered to be the most accurate modality to in vivo visualize the SCS [10].

## 3. Drug Delivery to the Suprachoroidal Space

### 3.1. Microcatheters

Using a full-thickness scleral incision, microcatheters can be inserted and guided into the SCS with a flashing diode. These procedures allow for precise targeting and visualization of the drug delivery site but require a skilled executor and an operating room setting [11,12]. Additionally, there is a risk of AEs such as SC hemorrhage, inadvertent choroidal penetration, and endophthalmitis, among others [11]. Currently, the iTrack microcatheter has been used to deliver medications like corticosteroids and anti-VEGF agents for diseases including glaucoma and neovascular age-related macular degeneration [13,14].

### 3.2. Hypodermic Needles

Standard small-gauge hypodermic needles can be used for SC injections [15,16]. Using a free-hand technique, the needle is inserted through the sclera, without a sclerotomy. This technique utilizes readily available, generic, and cost-effective materials but risks penetration of the vitreous humor, subretinal space, or subconjunctival area. Additionally, the technique requires significant precision, since a 30-gauge hypodermic needle (~300 μm outer diameter, bevel length > 1 mm) is being inserted through a ~500 μm thick sclera [17,18].

### 3.3. Microneedles

Hollow microneedles are microscopic applications with small-gauge needles only 0.7–1.0 mm in length [19,20]. The needles have an empty space inside filled with the treatment solution and are designed to only just penetrate the sclera, reaching the SCS. Microneedles are simple to use, less painful, minimally invasive, and can be used in an outpatient setting, making them the most promising form of SC administration.

When using SCS microneedles, it is recommended to first attempt the injection with a 900 µm needle, followed by a second 1100 µm needle if the SCS cannot be accessed. There was high acceptance of the SC injection procedure, where 84% of physician investigators did not find the SC injections more challenging than other ocular injection modalities [21]. Overall, the two microneedle lengths are effective and reliable in accommodating many different inter-individual scleral differences.

There has been ample development of novel microneedle designs, including new designs to deliver solid implants (as opposed to liquid formulations) [22] and other proprietary microneedles for DME (NCT06314217) [23]. Although suprachoroidal microneedles have received FDA approval, they must be dosed using a proprietary injector system. Recently, Katz and colleagues successfully created a lab-based SC microneedle for ex vivo and in vivo preclinical studies [24]. This microneedle, created using a 25-gauge microvitrectomy cannula, 34-gauge needle, and epoxy resin, had no issues with inadvertent IVT injections and very rarely needed slight repositioning. Although not appropriate for clinical studies, this innovative microneedle will improve accessibility and cost-effectiveness in pre-clinical settings, promoting further research expansion in this domain.

**Figure 1:**
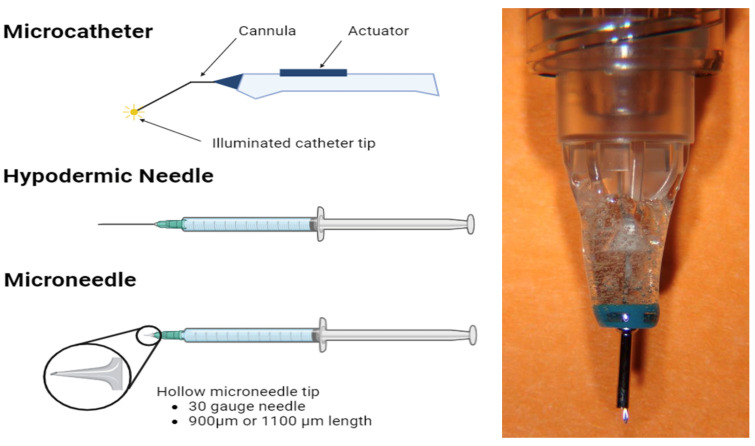
(**A**) Diagram of the three main suprachoroidal delivery systems: Microcatheter, hypodermic needle and microneedle. Created by Biorender. (**B**) Image of a lab-based microneedle for suprachoroidal delivery. Reprinted from Katz et al., 2024 [24]. Used under CC BY-NC-ND 4.0.

Figure 1 shows the three main suprachoroidal delivery systems: Microcatheter, hypodermic needle, and microneedle.

## 4. Ocular Administration Methods

**Figure 2:**
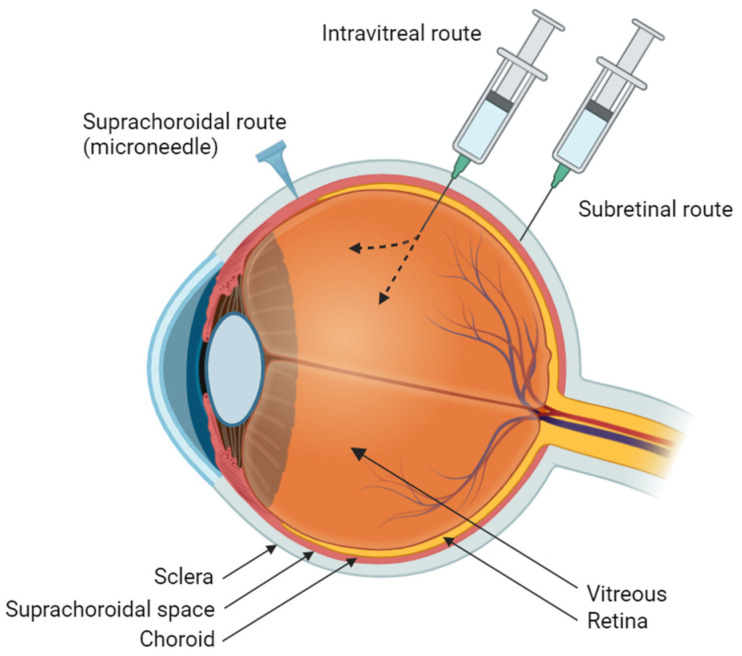
An overview of various ophthalmic medication administration routes. This figure illustrates a range of posterior segment administration methods in ophthalmic medicine, including suprachoroidal, intravitreal, and subretinal administration routes. Created with BioRender.com. Copyright © Kevin Y. Wu, 2024.

Routes of ocular medication delivery include topical, periocular, intravitreal, suprachoroidal, and subretinal. Intravitreal, suprachoroidal, and subretinal administrations are ideal for treating pathologies of the posterior segment of the eye, because they can bypass more anterior barriers, such as the tear film, cornea, and sclera (Figure 2).

### 4.1. Intravitreal Administration 

Intravitreal (IVT) injections are one of the most common in-office procedures across all specialties. They allow for direct delivery to the posterior segment of the eye and circumvents barriers such as the cornea, sclera, and blood–retinal barrier. IVT injections are convenient and applicable in an office-setting, bypassing many challenges with patient non-compliance [25]. However, potential adverse complications include increased intraocular pressure, intraocular inflammation, and cataract development [2]. Additionally, intravitreally delivered drugs often have short half-lives [26], requiring more frequent dosing.

### 4.2. Subretinal Administration

Subretinal (SR) administrations provide direct access to the outer retina but are invasive procedures that necessitate specialized surgical centers due to the need for pars plana vitrectomy and retinotomy [25]. Additionally, the procedure’s localized nature means that therapeutic benefits may be limited only to the area around the drug depot site [27].

### 4.3. Rationale for Suprachoroidal Administration

Suprachoroidal delivery allows for targeted delivery to the chorioretinal region, bypassing various ocular barriers for high bioavailability while compartmentalizing therapeutic agents away from the vitreous and anterior segments [25,27]. Additionally, SC injections are less invasive and have reduced risks of traumatic cataract and retinal tears [27]. Similar to IVT delivery, SC injections can be delivered in a clinic setting, but they have the advantage of bypassing ocular barriers such as the blood–aqueous barrier and outer blood–retinal and inner blood–retinal barriers, which limit drug penetration and efficacy [28]. Compared to SR delivery, SC injections can be done in an out-patient setting and provide a broader distribution along the retina [29].

SC injections are cost-effective and practical. A simulated 10-year US cost-effectiveness analysis based on the PEACHTREE trial found that suprachoroidal triamcinolone acetonide (SCTA) was cost-effective at USD 50,000 per quality-adjusted life-year gained compared to best supportive care for ME secondary to uveitis [30]. Similarly, a 5-year budget impact analysis found that SCTA could lead to lower-eye-related (inpatient, outpatient, emergency department visits), non-eye related, and pharmacy costs for the US healthcare system [31].

In terms of the patient experience, SC injections are safe and convenient. Acute eye pain, defined as pain occurring and resolving on the same day as the injection procedure, was reported in 3% of patients receiving SCTA [32], which is comparable to rates observed studies on IVT implants and anti-VEGF injections. Especially for patients that have previously undergone IVT injections, it is important to acknowledge potential differences, with potential temporary, mild discomfort due to SCS expansion. When counselling patients, it can be helpful to describe the sensation as a “pressure wave” [33]. Additionally, safety data from eight phase II and III clinical trials of SCTA show low rates of cataract and IOP elevation and no serious AEs involving lens injury, suprachoroidal hemorrhage, endophthalmitis, or retinal tear in patients receiving SC injections either alone or in conjunction with anti-VEGF [34].

SC injections can be slightly longer than IVT injections. SC injections require a three-step process: insertion of the microneedle, application of gentle pressure to form a seal, and a slow injection over 5 to 10 s, followed by maintaining the needle position for an additional 3 to 5 s to ensure proper delivery and limit reflux [33]​​​​. In comparison, IVT injections require a straightforward insertion of the needle into the vitreous cavity in a single motion. Additionally, potential for quadrant and needle switching or multiple attempts to inject at the chosen injection site, which can prolong SC procedure duration. To the author’s knowledge, no research has been done on patient-reported outcomes such as satisfaction, adherence or quality of life. However, clinical data has shown that SC injections offer the same convenience of in-office clinical use as IVT injections, with comparable safety profiles and improved efficacy [35] (Table 1).

## 5. Nanotechnology 

Nanotechnology includes any process that involves the design and creation of materials on the nanometer scale. This growing field’s potential in ophthalmology is particularly profound when applied to the posterior segment of the eye, given the complex anatomical and physiological barriers to drug delivery. Nanoparticles (NPs) can be designed to specifically target tissues, such as photoreceptor cells or retinal pigment epithelium. Additionally, nanocarriers minimize inadvertent side effects by protecting therapeutic agents against in vivo degradation and enhancing absorption, distribution, and bioavailability. Moreover, different nanocarriers can enhance drug release, increasing the window of therapeutic efficacy and reducing the dosage or frequency of administration. This is essential for treating posterior eye diseases as frequent intraocular injections can cause adverse side effects and reduce patient compliance [38]. Nanotechnology, in combination with suprachoroidal injections, shows significant potential in improving targeted drug delivery.

### 5.1. Nanomicelles

Nanomicelles are self-assembling nanoscale colloidal dispersions characterized by a hydrophobic core and hydrophilic shell. In aqueous solutions, amphiphilic molecules form spherical or cylindrical nanomicelles, allowing hydrophobic drugs to be loaded into their core, thereby enhancing solubility, stability, and targeted delivery. Conversely, in nonpolar solvents, these molecules form reverse nanomicelles with hydrophobic parts outward and hydrophilic parts inward, which is suitable for loading hydrophilic drugs [39].

Zhao and colleagues designed a nanomicelle drug delivery system made of copolymer EPC (nEPCs) to encapsulate aflibercept [40]. These aflibercept-loaded nEPCs penetrated the cornea in ex vivo porcine eye models and delivered a clinically significant amount of aflibercept to the retina in laser-induced choroidal neovascularization (CNV) murine models. Additionally, the inherent anti-angiogenic properties of nEPCs may enhance the anti-angiogenic effects of aflibercept. Nanomicelles are a promising avenue for targeted drug delivery to the posterior eye that circumvents traditional ocular barriers.

### 5.2. Nanoparticles

Nanoparticles (NPs), ranging from 1 to 1000 nm, can be composed of materials like metals, polymers, lipids, ceramics, or other substances [41]. They can be designed to encapsulate both hydrophobic and hydrophilic drugs, improving targeted delivery, efficient drug absorption, and controlled drug release. The most significant use of NP technology in the suprachoroidal space has been with the delivery of non-viral gene therapies, which are further discussed in Section 6.2.

#### 5.2.1. Polymer-Based Nanoparticles

Polymeric nanomaterials primarily consist of substances like polylactic acid (PLA), poly(lactic-co-glycolic acid) (PLGA), collagen, chitosan, and gelatin [41]. These materials are highly valuable due to their biocompatibility and biodegradability, making them ideal candidates for the foundational matrices for drug incorporation or encapsulation.

##### Natural Polymers

Natural polymers like cellulose, sodium alginate, hyaluronic acid, albumin, gelatin, and chitosan offer biocompatibility and minimal toxicity. Chitosan (CH) is a cationic polysaccharide derived from chitin and has favorable mucoadhesive retention times and minimal toxicity [42]. This enhanced retention time is due to chitosan’s capability to form strong molecular bonds between its positive amino groups and the negatively charged sialic acid residues in mucin [43]. Pandit and colleagues developed a chitosan-coated polylactide-glycolic acid NP (CS-PLGA) to deliver the bevacizumab for diabetic retinopathy [44]. Subconjunctival injections of the NPs demonstrated a favorable pharmacokinetic profile, with better permeability than the traditional drug solution. CS-PLGA NPs significantly reduced VEGF levels in the retina for up to 12 weeks compared to topical and IVT injections.

##### Synthetic Polymers 

Synthetic polymers commonly used in NPs include polylactic acid (PLA), poly(lactic-co-glycolic acid) (PLGA), poly(ε-caprolactone) (PCL), polymethacrylic acid (PMAA), and polyacrylamide. Compared to natural polymers, synthetic polymers offer greater control and customizability but are usually less biocompatible and may be more likely to provoke an immune response. Additionally, synthetic polymers usually provide more consistency in manufacturing but often have higher production costs due to the potential complexity of their synthesis and purification processes [45].

PLA and PLGA have been used for drug delivery systems. Zhang and colleagues designed a bevacizumab-encapsulated PLGA nanoparticle that prolonged the residency and drug duration in the vitreous and aqueous humors [46]. Bevacizumab-encapsulated PLGA had no significant toxicity in vitro or in vivo. Moreover, the encapsulated PLGA was more effective than bevacizumab alone in inhibiting VEGF-mediated endothelial cell proliferation and demonstrated enhanced anti-angiogenic effects. Similarly, another study used a solid/oil/water emulsification technique to create microspheres containing bevacizumab with PLGA/PCADK as a carrier vessel [47]. These microspheres had a prolonged release of over 50 d in both in vivo and in vitro settings.

Additionally, Prieto and colleagues developed a novel sustained-release formulation of dexamethasone involving laponite [48]. This dexamethasone-laponite nanoformulatoin was injected both intravitreally and suprachoroidally into 30 rabbit eyes. Both administration methods were well tolerated, with SC injections causing only transient conjunctival hyperemia and epithelial defects, while IVT injections led to early cataract formation. The dexamethasone–laponite formulation achieved higher and more prolonged dexamethasone levels in the vitreous humor compared to conventional dexamethasone solutions, with significant intraocular retention time. SC administration sustained detectable dexamethasone levels in the choroid–retina unit and sclera for up to 24 weeks. The study concluded that the dexamethasone-laponite formulation is a biocompatible and effective sustained release system suitable for treating posterior-segment eye diseases, offering potential benefits such as improved drug bioavailability, reduced re-administration frequency, and enhanced patient compliance and outcomes.

#### 5.2.2. Lipid-Based Nanoparticles

In contrast to polymeric NPs, lipidic formulations are known to be less stable for sustained drug release. Lipid NPs are formed by solid lipids (SLNs) and liquid lipids (NLCs). At body temperature, solid lipids form a highly structured crystalline lattice that allows for a sustained drug release. SLNs consist of the majority of lipid-based nanoparticles, and lipid screening is frequently used as a tool to choose a lipid that is compatible with the active compound. A promising treatment for dry age-related macular degeneration (AMD) is pentamethyl-6-chromanol, an antioxidant with potent protective effects for the retinal pigment epithelium (RPE). Arta and colleagues utilized high-shear homogenization to create SLNs loaded with pentamethyl-6-chromanol [49]. In vitro tests showed that these SLNs suppressed reactive oxygen species (ROS) and prevented the accumulation and destabilization of other oxidant molecules like lysosomal ox-LDL.

Nanostructured lipid carriers (NLCs) possess a solid lipid core composed of both solid–liquid and liquid–liquid interfaces. This lipid-based matrix has a lower melting point than the original solid lipid. They are generally less favored than SLNs but are also being investigated for primarily topical administration in the pre-corneal segment of the eye.

#### 5.2.3. Metal-Based Nanoparticles

In addition to nanocrystals, inorganic nanoparticle packaging shows promise in conjugation with suprachoroidal deliveries. Compared to organic nanomaterials, metal and metal nanomaterials have unique biomedical properties, high stability, and resistance to degradation, but poor biodegradability.

Tzameret and colleagues developed a novel suprachoroidal iron oxide/human serum albumin NP injection that demonstrated potential as an intraocular drug delivery method [50]. Superparamagnetic iron oxide is non-toxic and biodegradable, and due to its iron content it can be tracked in vivo by magnetic resonance imaging. Additionally, human serum albumin is a versatile protein carrier for drugs that can easily be used to conjugate various biomolecules. Together, this nanoparticle demonstrated widespread distribution and residence in the posterior segment of a rat model of retinal degeneration. These results are promising and may provide significant utility as an extended-release posterior drug delivery method with translational utility in animal studies.

In addition to its significant diagnostic potential, metal nanoparticles show encouraging therapeutic applications. Gold nanoparticles (AuNPs) are particularly promising for ocular drug delivery due to their unique anti-angiogenic and anti-inflammatory properties. For example, one study has demonstrated that AuNPs effectively inhibit VEGF- and IL-1β-induced growth and migration in retinal pigment epithelial cells by blocking the Src kinase pathway [51]. This positions AuNPs as potential therapeutic agents for conditions like proliferative vitreoretinopathy. Additionally, Pereira et al. found that AuNPs reduce TNF-α and myeloperoxidase levels in endotoxin-induced rat uveitis, indicating their role in mitigating inflammation and oxidative damage via the TLR4-NF-κB pathway [52]. Combining nanotechnology with suprachoroidal delivery offers customizable and encouraging treatment alternatives, potentially reducing the frequency and dosage of intraocular injections while ensuring widespread distribution and prolonged residence in the posterior segment.

### 5.3. Liposomes 

Liposomes are vesicles with lipid bilayers encapsulating a hydrophilic core. Composed mainly of phospholipids, cholesterol, and wax lipids, they mimic cell membranes, thus ensuring excellent biosafety and tissue compatibility. Liposomes are the first nano drug delivery system to be successfully translated into clinical application, with their inaugural approval from the US Food and Drug Administratoin in 1995 [53]. Since then, a wide range of commercially available liposomal products have been used to treat a myriad of disorders affecting the posterior ocular segment, with a particular emphasis on AMD. One example is Visudyne, a photoactivatable liposomal formulation of verteporfin indicated for the treatment of sub-foveal CNV in AMD [54].

Additionally. Altamirano-Vallejo and colleagues developed a liposomal formulation of tretinoin, which showed higher safety compared to conventional IVT steroid injections [55]. In vivo studies indicated maximum TA concentrations in the retina and vitreous humor 12 h after topical application, effectively delivering the drug without significantly impacting cell viability or intraocular pressure. Similar formulations have been used for treating macular edema. Li et al. created tretinoin-CHL, a liposome encapsulated with chitosan, with an average size of 135.46 ± 4.49 nm [56]. This nanosized liposome demonstrated a sustained release profile, physical stability, and no significant toxicity and adequate penetration into the posterior eye despite its topical administration.

### 5.4. Hydrogels

Hydrogels are three-dimensional cross-linked networks with high water content that are formed by either bonding hydrophilic polymer chains together or polymerizing water-soluble monomers [57]. They have unique properties such as excellent biocompatibility, tissue-like flexibility, and extensive customizability.

Deng and colleagues created a supramolecular hydrogel by linking antioxidant 3,5-dihydroxybenzoic acid (DHB) and non-steroidal anti-inflammatory drugs to self-assembling peptide segments (GFFYD) [58]. This hydrogel’s viscoelasticity helps resist tear washout, prolonging pre-corneal residence time. In both laboratory and rabbit uveitis models, the DHB0-FFYD hydrogel has demonstrated antioxidant and anti-inflammatory properties. Similarly, another study developed chitosan hydrogel eye drops for a non-invasive uveitis treatment [57]. Loaded with the anti-TNF-alpha antibody adalimumab, this adalimumab-loaded hydrogel eye drops had superior penetration and clinical efficacy compared to adalimumab alone.

Within the suprachoroidal space, recent animal studies have successfully delivered PLGA and polycaprolactone dimethacrylate (PCM) and hydroxyethyl methacrylate (HEMA) gel networks into the suprachoroidal space [59,60]. In Hackett and colleagues’ study, their PLGA hydrogel successfully delivered microspheres of acriflavine into the suprachoroidal space [60]. After 28 d, rabbits maintained therapeutically relevant levels of acriflavine throughout the eye that significantly reduced the development of laser-induced choroidal neovascularization. The gel was well-tolerated with normal-appearing retinas, no increases in intraocular pressure, and normal retinal histology. Similarly, a PCM and HEMA gel demonstrated sustained bevacizumab release for four months while maintaining its stability and VEGF-binding activity [59]. This light-activated PCM and HEMA gel is well suited to in situ gel formation, showing significant potential for sustained protein delivery. Combining hydrogel and nanotechnology allows for a high degree of customizability in ocular delivery.

Nanotechnology shows great promise for the posterior segment of the eye, overcoming anatomical and physiological barriers to drug delivery and enhancing treatment effectiveness. NPs can be engineered for targeted drug delivery, minimizing side effects and enhancing absorption, distribution, and bioavailability. Nanocarriers, such as nanomicelles, lipid-based NPs, and polymer-based NPs, can improve drug release, reducing the need for frequent administrations. These technologies show great potential when combined with suprachoroidal delivery, a minimally invasive method that allows higher drug concentrations to reach the retina and choroid, thus offering a promising alternative for treating posterior ocular diseases.

## 6. Gene Delivery to the Suprachoroidal Space

Gene therapy involves delivering genetic material into cells to compensate for defective genes or produce a beneficial protein. It is a rapidly advancing field, particularly in ophthalmology, where the eye and retina serve as an ideal target due to their small size, natural compartmentalization, and immune-privileged nature. Additionally, ophthalmology offers advanced diagnostic capabilities, the presence of an in-built contralateral control, and direct routes of administration [61]. Various viral and non-viral vectors provide different advantages and disadvantages, with most research focused on viral gene therapies (Table 2). SC delivery is an emerging avenue for gene therapies, as an in-office procedure that has good penetration and greater posterior segment coverage. Despite these advantages, SC delivery also presents challenges such as rapid clearance due to the proximity of the choriocapillaris, systemic exposure risks, and potential immune responses from preexisting neutralizing antibodies. Further research is needed to fully realize benefits and address existing challenges.

### 6.1. Viral Gene Therapy

Viral gene therapies leverage the viruses’ ability to introduce new DNA into host cells and require delivery of one of three viral vectors: adeno-associated viruses (AAV), adenovirus vectors, or lentiviral vectors. The AAV is a protein shell surrounding a small, single-stranded DNA genome that relies on co-infection of other viruses, mainly adenoviruses, to replicate [63]. Adenovirus vectors are larger, double-stranded DNA viruses (~36 Kb) encased in a protein shell. These vectors are less commonly used for gene therapies, as they are highly immunogenic and can be beneficial for vaccine applications, but problematic for chronic treatments [64,65]. Finally, lentiviral vectors are composed of single-stranded RNA as opposed to AAV and adenovirus vectors, which are composed of DNA. Lentiviral vectors integrate into the host genome but carry risks of mutagenesis and increased risks of adverse reactions [66].

The AAV is one of the most widely investigated and utilized gene therapy vehicles, and has been consistently shown to be safe and efficacious for long-term transgene expression [63]. However, despite the many advantages of AAV vectors, the limited packaging capacity of ~4.8 Kb remains a challenge for large transgenes [67]. Ophthalmic gene therapy research has focused on AAV2, AAV5, AAV8, and AAV9 vectors, with AAV8 considered the reliable choice for SC injections. Animal studies [37,68] have shown lower systemic humoral immune response from SC AAV8 as compared to IVT AAV8. However, intraocular inflammation was more pronounced with SC delivered AAV8 compared to subretinal. Furthermore, there was a noted decrease in transgene expression after 2 to 3 months, potentially due to cellular damage and increased local inflammation.

RGX-314 is an AAV8 vector that encodes for a monoclonal antibody fragment that inhibits VEGF activity. Ding and colleagues’ (2019) murine model demonstrated similar expression of RGX-314 in the retina, RPE, and choroid with both SC and SR delivery. Both routes showed significant suppression of VEGF-induced vasodilation and vascular permeability [15]. AAVIATE (NCT04514653) is a phase II dose escalation trial that will compare RGX-314 with monthly ranibizumab in patients with neovascular age-related macular degeneration (nAMD) [69,70]. While ongoing interim data have shown good tolerability with meaningful reduction in treatment burdens with no difference in outcomes for patients with detectable neutralizing antibodies. Within the third cohort, 67% of RGX-314 treated patients (n=10) did not require anti-VEGF injections at six months.

Furthermore, ALTITUDE (NCT04567550) is an ongoing phase II clinical trial evaluating the use of RGX-314 in patients with diabetic retinopathy with or without center-involved DME [71]. Preliminary results are encouraging, as SC RGX-314 has been well-tolerated so far with noted improvements in disease severity in the treatment group [72]. Notably, there are also two ongoing studies, ATMOSPHERE (NCT04704921) and ASCENT (ASCENT), studying the subretinal delivery of RGX-314 for nAMD.

Excitingly, Luo and colleagues (2024) have designed a novel AAV vector (AAVv128) that exhibits significant transduction efficiency and broad distribution in a CNV model of nAMD [73]. At 35 d, SC delivered AAVv128 demonstrated the complete inhibition of Grade IV lesions, compared to 31.25% in the AAV8-treated group. Additionally, AAVv128 had a four-fold increased rate of transduction in the photoreceptors, RPE, and horizontal cells compared to AAV8. The development of the AAVv128 vector holds great promise for advancing ocular gene therapy.

Lentiviral vectors offer an interesting different avenue of ocular gene therapy. Currently, AAVs are generally favored to avoid an immunogenic response. Nevertheless, AAVs are limited by their therapeutic cassette, which is limited to less than 5 kb, and pre-existing immunity is still a barrier for many capsids [67]. Current research on lentiviral vectors is focused on developing envelope modifications that help redirect lentiviral vectors’ affinity for specific cell types. For instance, lentiviral vectors have shown success in targeting the RPE or trabecular meshwork [66,74,75], but controversies exist regarding their efficacy in transducing photoreceptors [76,77].

This emerging research has also begun to utilize SC injections. Currently, an ongoing phase I clinical trial (NCT05099094) will assess the SC delivery of BDS311 [78]. This integration-deficient lentiviral vector which will carry a VEGFA antibody gene to the RPE cells in patients with refractory retinal and choroidal neovascularization diseases, such as nAMD, diabetic ME, and retinal vein occlusion ME. Recruitment is ongoing with no published data yet.

Many current viral gene therapies focus on AAV technology via SR or IVT administration. SC deliveries are an emerging avenue, producing a more homogenous delivery of transgenes, avoiding risks of invasive surgery, and less systemic escape and humoral immunity [37,68]. Ongoing phase II trials will provide pivotal data on the efficacy of viral gene therapies in retinal diseases, and the different administration modalities.

### 6.2. Non-Viral Gene Therapy

Suprachoroidal (SC) non-viral gene delivery is a newly emerging approach circumventing the need for viral vectors. Non-viral gene therapies have been less extensively studied due to lower transduction efficiency and expression levels. Delivering hydrophilic molecules like DNA is challenging, given that cells are also negatively charged. Once the gene does enter the cell, the DNA is often degraded by endosomes. The simplest of non-viral approaches, which is the physical delivery of naked plasmid DNA, is not well-suited for SC delivery. Although successful in preclinical settings, the delivery of naked DNA often needs invasive surgery to place microelectrodes near the target tissue making ex-vivo application difficult [62,79,80]. Additionally, naked DNA has limited uptake due to quick degradation [81]. To address these challenges, nanotechnologies offer novel opportunities in the modification and delivery of gene therapies.

Chemical forms of non-viral gene delivery include different NPs as carriers to introduce circular, double-stranded plasmid DNA into target cells. Advantages of NPs include accommodating larger DNA sizes and lower immune responses. Additionally, NPs do not risk creating self-replicating agents and are much more cost-effective than viral vectors [82,83]. Peptide-based NPs are considered the best due to their ability to target specific cell receptors, induce minimal immune response, and allow for higher doses [84]. Additionally, polymer-based NPs are being increasingly utilized due to their versatile structures and easy synthesis [16,85,86]. Several liposomal NP compounds have been tested in the ocular context using IVT and SR routes, but to the best of the authors’ knowledge, SC administration routes remain unexplored [87,88].

SC injection of nanoparticles shows potential for the treatment of various retinal diseases. Kansara et al. [89] demonstrated that SC and SR rod- and ellipsoid-shaped luciferase protein DNA NPs in rabbits and non-human primates were well-tolerated, with sustained luciferase activity up to 22 d in the retina, choroid, and RPE. These results were replicated in a follow-up study comparing [90] SC ellipsoid-shaped, SC rod-shaped and SR rod-shaped DNA nanoparticles (DNP) in rabbits. There were no electroretinography differences between SC and SR delivery. SC ellipsoid DNPs had a mild to moderate immunogenic response, while SC rod DNPs showed no intraocular inflammation or toxicity.

Non-viral gene therapy allows the delivery of larger genes. In 2021, Kansas et al. [91] compared SC and IVT injections of DNPs containing plasmid DNA encoding for human myosin7A (hMyo7A), a large protein with molecular mass of 254 kDa [92], in rabbits. SC injections were better tolerated and equally effective in gene transfection than IVT injections. This study demonstrated promising potential in future clinical applications, facilitating the delivery of larger genes with lower immunogenic response. Nanoparticles allow for repeated gene therapy injections and can transfer larger genes, unlike AAV vectors. However, non-viral gene therapies can result in variable expression and have variable risks of immunogenic responses.

## 7. Pharmacokinetics

Pharmacokinetics involves how the body interacts with drugs, including their uptake, distribution, and elimination. For SC injections, key considerations include drug distribution within the SCS (anterior vs. posterior), across the eye (anterior vs. posterior segments, within specific ocular layers), and clearance.

### 7.1. Distribution

The SCS has anatomical boundaries that help confine administered drugs and reduce systemic exposure. Scleral thickness and tight adherence to the ciliary body at the scleral spur occlude the suprachoroidal and supraciliary spaces anteriorly. This occlusion ensures that fluid introduced in the pars plana region will expand the SCS and flow posteriorly [7]. This posterior flow is further enhanced by a natural hydrostatic pressure differential (anterior: 0.9 +/− 0.2 mmHg less than IOP, posterior: 3.7 +/- 0.4 mmHg, p<0.001) [93]. This differential drives uveoscleral outflow, enhancing posterior drug distribution in the SCS.

Animal studies show that SC injections achieve higher drug concentrations in the chorioretinal region than IVT injections. An ex vivo study on porcine eyes demonstrated successful injection and compartmentalization of red fluorescent sulforhodamine B between the sclera and choroid [19]. This was further validated in an in vivo rabbit model, which demonstrated 10- to 100-fold-higher drug concentrations in the choroid and retina compared to the IVT injections [20]. Similarly, Tyagi et. al (2012) demonstrated that SC injections caused 36- and 25-fold-higher drug concentrations in the chorioretinal region (*p* = 0.001) than posterior subconjunctival and IVT, respectively [36].

Other small molecule suspensions have been evaluated in rabbit models. SC injections delivered 12 times more triamcinolone acetonide (TA) to the sclera, retina, and RPE compared to IVT delivery [94], with negligible amounts detected in the anterior chamber. When delivered suprachoroidally, small molecules like axitinib and A01017 showed the highest concentration in the chorioretinal region [29,95]. Negligible amounts were detected in the vitreous humor post-injection [95].

The volume of injectate also impacts the distribution of fluid in the SCS. In an ex vivo rabbit model, increasing the volume of injectate increased the overall circumferential coverage of the SCS. Suprachoroidal injections of 75 µL or more of fluorescein were able to cover more than 50% of the choroidal surface [96]. Interestingly, the increase in fluid volume from 25 to 150 µL correlated with increased SCS fluid coverage as opposed to the thickness of the SCS near the injection site. This suggests that increased injectate volume will increase coverage, and therefore therapeutic benefit, as opposed to just localized expansion near the area of the single injection.

Additionally, injections into multiple quadrants of the eye have been shown to increase overall distribution. Nork and colleagues performed two SC injections of sodium fluorescein (50 µL each) in the superotemporal and inferonasal quadrants, which led to greater SCS coverage than a single injection [97].

### 7.2. Clearance

**Figure 3:**
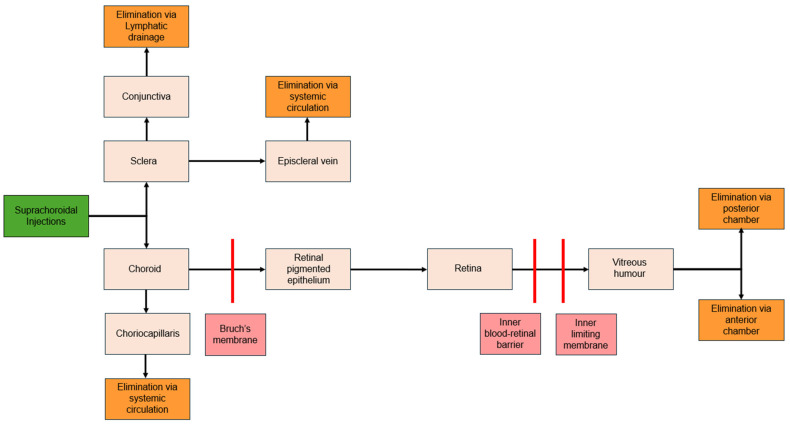
This figure illustrates the anatomical distribution and clearance pathways within suprachoroidal space. The red lines and red boxes highlight specific anatomical barriers that restrict fluid movement and protect the retina from systemic influences. Orange boxes represent different elimination pathways.

SCS visualization shows that the SCS expands in a dose-dependent manner and returns to baseline once the injected fluid clears [98]. Most molecules in the SCS are cleared into systemic circulation by the choriocapillaris within an hour or convected trans-sclerally over several hours [99] (Figure 3).

The clearance rate of substances from the SCS is influenced by particle size, viscosity, injected volume, and particle solubility. Choriocapillaris fenestrations can clear particles sized 6 to 12 nm [100], with very small particles (20 nm to 10 µm) and very large macromolecules (2 MDa) showing slower clearance rates [20,99,101]. Additionally, higher-viscosity and low-solubility solutions have been shown to result in slower SCS distribution and clearance [99,102]. Chiang et al. evaluated clearance kinetics and particle size where fluorescent molecules of varying molecular weights were injected into the SCS. Increasing molecular weight results in longer clearance times; While fluorescein (320 Da) was cleared within 12 h, fluorescent dextran (2 MDa) was observed for up to four days [99]. Injection volumes also have varying impacts on SCS expansion depending on the injectant’s formulation [19,98,101]. These different formulation adjustments give researchers the means to optimize therapeutic effects while minimizing systemic exposure and side effects.

Different polymers can also extend the lifetime of injections materials in the SCS. For example, RGD peptide hydrogels were found to be biocompatible and biodegradable in rabbits. The hydrogel could be clearly identified by ultrasound and confirmed to be non-toxic to retinal vessels via fluorescein angiography. There were no histological abnormalities in the retina, choroid, or surrounding tissues. Moreover, the hydrogel had a lifetime of 14.3 ± 3.3 d in the SCS and 25.7 ± 3.3 d in the vitreous cavity [103]. Another promising novel vehicle is a light-activated polycaprolactone dimethacrylate and hydroxyethyl methacrylate gel network, which maintained stable VEGF-binding activity with the sustained bevacizumab release for four months [59]. Similarly, Hackett et al. incorporated acriflavine, an angiogenic factor HIF-1 inhibitor, into a poly-lactic-co-glycolic-acid (PLGA) for SC and IVT injections. Alone, acriflavine is a small molecule that clears rapidly from the eye in solution form. However, PLGA loading demonstrated durable suppression of choroidal neovascularization in rats and mice for at least 18 weeks [60] with a particle size of approximately 7 µm.

Animal studies show that SCS injections of aqueous drugs are cleared significantly faster than IVT injections. For example, bevacizumab levels were not measured after 7 days when injected into the SCS, compared to 30–60 d with IVT injections [12]. Additionally, ketorolac was eliminated faster from the SCS with a half-life of 1.19 h versus 3.09 h for IVT injections [104].

## 8. Clinical Applications 

This section, we present a detailed review of the use of SC injection for treating various ocular diseases. For further details on each referenced study, please refer to Table 3.

### 8.1. Macular Edema 

#### 8.1.1. Macular Edema Due to Uveitis

Uveitis, the inflammation of the uveal tract, is classified into anterior, intermediate, posterior, or panuveitis based on the site of inflammation. A significant complication is uveitic macular edema (UME), characterized by fluid accumulation within the retinal layers or subretinal space due to inflammation-induced breakdown of the blood–retina barrier. UME leads to vision loss in about one-third of posterior uveitis cases [126].

Diagnosis of UME in uveitis patients is confirmed using optical coherence tomography (OCT) and fundus fluorescein angiography (FFA). These tools also help detect subclinical macular edema, allowing for early intervention to prevent long-term ocular morbidity. Uveitis is responsible for about 20% of legal blindness cases in developed countries [127].

Management of uveitis first involves ruling out infectious causes and malignancies (i.e., masquerade syndromes). Once non-infectious uveitis is confirmed, UME treatment primarily involves corticosteroids. Systemic steroids, such as oral prednisolone (1–2 mg/kg), are used for bilateral uveitis or cases unresponsive to peri-ocular injections. Peri-ocular injections of triamcinolone acetonide (2–4 mg/0.1ml) or methylprednisolone acetate (40–80 mg) are alternatives when systemic steroids are contraindicated. Intravitreal steroids, including triamcinolone (2–4 mg/0.1ml) or implants like fluocinolone (590 micrograms) and dexamethasone (700 micrograms), are options for recalcitrant cases. Though the role of topical NSAIDs in controlling UME is not well-established, drugs like bromfenac and nepafenac may enhance steroid effectiveness [126,128].

Suprachoroidal injections have shown significant clinical benefit in uveitis. The first and only FDA-approved treatment using this approach is the triamcinolone acetonide injectable suspension (Xipere) for macular edema secondary to non-infectious uveitis [35]. Traditional treatments like IVT and local corticosteroid therapy are associated with side effects such as high IOP, glaucoma, and cataract development [128]. The SCS provides a novel delivery method that limits anterior segment exposure, potentially circumventing corticosteroid-related side effects.

**Figure 4:**
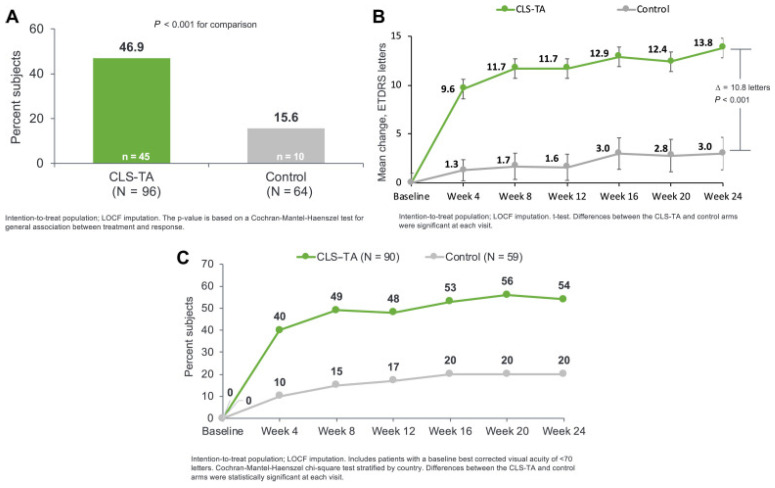
Comparison of changes in best-corrected visual acuity after SC injection of 4.0mg of triamcinolone acetate versus a sham injection from the PEACHTREE trial [129]. (**A**) Bar graph showing percent of patients gaining 15 or more Early Treatment Diabetic Retinopathy Study (ETDRS) letters from baseline at Week 24. (**B**) Graph showing mean ± standard error of the mean change from baseline in best-corrected visual acuity in ETDRS letters read at each visit. (**C**) Graph showing percent of patients reading 70 or more ETDRS letters (approximately 20/40 or better) at each visit. CLS-TA = triamcinolone acetonide formulation; LOCF = last observation carried forward. Used under CC BY-NC-ND 4.0.

The pivotal PEACHTREE (NCT02595398) trial investigated SC delivery of CLS-TA for non-infectious uveitis with macular edema [129]. The double-blinded study included 160 eyes randomly assigned to either 4.0 mg of SCTA at day 0 and week 12, or a sham injection. The study was broadly inclusive, including inactive uveitis, active uveitis, and uveitis of any anatomic location. Additionally, patients could remain on systemic corticosteroid and/or immunomodulatory therapies. At 24 weeks, 47% of CLS-TA patients achieved significant BCVA improvement, gaining 15 or more EDTRS (16% sham, *p* < 0.001), as seen in Figure 4. Additionally, there was substantial reduction in CST thickness (-153 µm in the SCTA group vs. -18 µm in the control group; *p* < 0.001) and ME resolution (CST < 300 mm; 53% in the SCTA group vs. 2% in the control group). Additionally, no serious treatment-related adverse events were reported. Elevated intraocular pressure and cataract adverse events were similar between both groups (Elevated IOP: 11.5% SCTA and 15.6% control, Cataract: 7.3% and 6.3% respectively).

Further post hoc analyses found that the benefits demonstrated by PEACHTREE were consistent across age groups (≤ 50 or >50 years), concurrent baseline systemic corticosteroid use, and uveitis subtypes (anterior, intermediate, posterior, and panuveitis) [105,106,130]. MAGNOLIA (NCT02952001) was a non-interventional extension study involving the 33 PEACHTREE patients who did not receive rescue therapy [131]. The median length of time until rescue therapy was needed was 257 d in the study arm and 55.5 d in the control arm (*p* < 0.001). Additionally, inflammation measures, such as anterior chamber cells and flares, were stable or improved.

Henry et al. (2022) conducted AZALEA (NCT03097315), a 24-week open-label phase III clinical trial that evaluated the safety of SC-TA in 38 patients with non-infectious uveitis with or without macular edema [132]. Similar to PEACHTREE, patients received two 4.0 mg SCTA injections, one at day 0 and one at Week 12. The results showed a favorable safety profile with no serious ocular AEs and 7 treatment related AEs (18.4%). The most frequently reported AEs were pain (7.9%), IOP rise >10 mmHg (15.8%), and IOP > 30 mmHg (5.3%) being most common. Only 10.5% of patients (*n* = 4) needed rescue therapy with improved signs of inflammation. Mean BCVA improved at all post-baseline visits, measuring 75.9 (SD 15.82) at Week 24. In subjects with a baseline BCVA of ≤ 80 letters, 63.0% of patients had a gain of at least 5 letters at Week 24.

Yeh and colleagues’ safety analysis of PEACHTHREE and AZALEA confirmed SC delivery efficacy [108]. However, direct comparisons with other local administration methods are needed (i.e., periocular TA injections, IVT TA injections and IVT dexamethasone implants). These other treatment modalities were directly compared in the phase III trial (POINT, NCT02374060), which found that IVT TA and IVT dexamethasone implants were superior to periocular TA [133]. Although cross-trial comparisons have limits, the proportion of eyes with a ≥ 20% reduction in CST at 24 weeks was 73% for IVT TA, 74% for IVT dexamethasone implants, and 61% for periocular TA, compared to 67% of SCS-TA patients in Yeh’s analysis. Additionally, mean gain in BCVA was 9.6 ETDRS letters, 9.2 letters, and 4.1 letters respectively, compared to 13.9 letters with SCS-TA treatments.

With the rollout of SCS-TA in clinical practice, retrospective data offer new insights into its practical effectiveness. Zhou and colleagues’ study indicates that SCS-TA was beneficial in a real-world multi-provider single-center setting, with minimal IOP elevations, even in patients with a history of steroid response [107]. Further studies in practical non-controlled settings will provide important shed light on real-world efficacy and safety.

Numerous trials have demonstrated the safety and efficacy of SC corticosteroid injections and paved the way for further investigations in other conditions, such as diabetic ME, AMD, retinal vein occlusion (RVO), and choroidal melanoma.

#### 8.1.2. Diabetic Macular Edema

A complication of poorly managed diabetes mellitus (DM), diabetic macular edema (DME) has now become the leading cause of vision impairment in diabetic patients. Epidemiological studies show that approximately 30% of diabetic patients worldwide have vision-threatening DR, with 3.8% in the U.S. suffering from DME [134].

DME is characterized by hard exudates and macular edema resulting from damage to the retinal microvasculature, which can be detected through clinical examination or optical coherence tomography (OCT) [134]. Historically, focal laser photocoagulation was the primary treatment for DME. However, recent clinical evidence supports intravitreal (IVT) anti-VEGF injections as the first-line therapy, with corticosteroids as a secondary option for those who do not respond adequately [135,136].

The standard first-line treatment for DME involves IVT injections of anti-VEGF agents, such as ranibizumab, aflibercept, and bevacizumab [134]. Despite their effectiveness, IVT treatments require frequent injections and carry potential adverse effects. Second-line treatments include corticosteroids such as TA, with similar ocular AEs such as raised IOP and cataract development [134].

SC delivery of TA (SCTA) is a promising new alternative to traditional IVT treatment. The HULK trial (NCT02949024) demonstrated the superiority of combined SCTA and IVT aflibercept against SCTA alone [137]. After 6 months, the combination treatment group had greater visual acuity gains, larger CST reductions, and no serious AEs. A subsequent phase II trial, TYPEE (NCT03126786), compared SCTA and IVT aflibercept therapy to IVT aflibercept alone [138]. TYPEE showed that mean BCVA changes did not differ between the two groups (+ 12.3 vs. + 13.5 letters, *p* = 0.664), however there were greater CST reduction in the combination group (-226.5 μm vs. -176.1 μm, *p* = 0.035). Additionally, the SCTA and aflibercept combination group received fewer treatments, with no treatment-related serious AEs.

Moreover, a recent animal study showed favorable results in the SC delivery of aflibercept in a murine CNV model [112]. Both SC and IVT injections of aflibercept significantly reduced leaky CNV lesions by day 3 (SC: 49.8%, *p* < 0.05; IVT: 56.8% (*p* < 0.01). However, SC’s efficacy wore off on Day 7, with a non-significant 76.8% reduction in leakage area compared to the control (*p* = 0.14).

Studies have also compared SC and IVT delivery of bevacizumab. In Fazel et al.’s 2023 trial (IRCT20200314046761N1), 66 eyes were randomized to either a combination of SCTA with IVT bevacizumab or bevacizumab monotherapy [110]. Combined therapy offered superior improvements in visual acuity, reduced retinal thickness and macular volume. Similarly, Anwar’s 2022 observational study demonstrated SCTA’s superior effects in improved visual acuity, mean CST reduction, and efficacy compared to bevacizumab [139].

In addition to the ongoing research on VEGF inhibitors and corticosteroids, there is ample research and development in different proprietary SC delivery devices, including microcatheters (NCT05512962) [111] and microneedles (NCT06314217) (51). OXU-001 is a cutting-edge sustained-release dexamethasone formulation designed to be injected into the SCS using a proprietary Oxusphere polymer system [111]. OXEYE (NCT05697809) is an ongoing phase II trial designed to compare the SC delivery of OXU-001 with an IVT dexamethasone implant in patients with DME [113,140]. If successful, this technology has the potential to deliver long-lasting treatments for up to 12 months through a minimally invasive approach. Early results are expected in late 2024.

A recent retrospective case series by El Rayes and colleagues (2023) has shown strong potential in the SC delivery of a fluocinolone acetonide implant [114]. The case series’ results are promising, with significant gains in median BVCA (pre-operative 0.07 to post-operative 0.15, *p* = 0.02), a 25% CST reduction from baseline (462 to 348 μm; *p* = 0.023) at four weeks post-injection and a 20% CST reduction (363 μm; *p* = 0.045) at 24 weeks. Additionally, 20% of patients developed grade I nuclear sclerosis by 12 months, while 50% of patients had a transient rise in IOP < 10 mmHg that resolved with antiglaucoma drops. In comparison, rates of cataract development range from 25% to 84.5% in clinical trials that investigated IVT deliveries (FAME, RESPOND, and PALADIN) [141,142,143]. Additionally, all three of the previously mentioned trials needed laser trabeculoplasty (0.8% to 2%) or glaucoma surgery (1.5% to 8.1%) to lower the IOP of its patients [141,142,143]. Overall, data shows that SC delivery is potentially equally as effective and safer than IVT delivery. Future studies necessitate larger sample sizes and concurrent comparisons with other treatment modalities.

Another key research area is in small molecule therapies, compounds that typically weigh less than 800–900 Daltons. These molecules are ideal due to their small size and simple chemical structure, which facilitate easy synthesis and formulations into affordable medications.

Like VEGF, integrins are a part of the angiogenic cascade and have become a recent treatment target in vitreoretinal disease. Integrin antagonists inhibit neovascularization, reduce inflammation, and prevent retinal vascular leakage in animal studies [144,145]. Recently, SC delivery of CLS-301, a small molecule integrin antagonist, has demonstrated potential in an in vivo rabbit model by Kansara and colleagues [115]. CLS-301 was well-tolerated with no overt toxicity or intraocular inflammation. Moreover, mean CLS-301 levels in the central retina were well-maintained, measuring 30- to 550-fold higher than in-vitro IC50 values. By Day 112, mean drug levels in the central and peripheral retina were 1–2 orders of magnitude higher than in vitro values. Low drug levels were detected in plasma, aqueous humor, and vitreous humor samples.

Plasma kallikrein-kinin inhibitors are another small molecule that have been shown to improve retinal vascular abnormalities and reduce retinal vascular permeability in rat models [146]. A recent 12-week preclinical study run by Clearside Biomedical investigated BCX4161, a plasma kallikrein inhibitor, in rabbit models [116]. Bilateral SC injections of BCX4161 demonstrated sustained localization of BCX4161 in the chorioretinal region with promising bioavailability and tolerability. The retina showed mean drug concentrations that were 10 to 100 times higher than those in the vitreous humor. Moreover, low levels of BCX4161 were detected in samples of aqueous humor and plasma.

As previously mentioned, SC viral gene therapies show potential, with a phase II clinical trial (ALTITUDE, NCT04567550) currently enrolling patients for the evaluation of RGX-314 therapy in patients with diabetic retinopathy with or without DME [71].

Overall, SC delivery of SCTA has promising advantages compared to conventional IVT modalities. Further research into dosing and comparative effectiveness is needed. Additionally, novel research into small molecule suspensions, such as plasma kallikrein and integrin inhibitors, and gene therapies are underway, with promising preclinical potential.

#### 8.1.3. Macular Edema Due to Retinal Vein Occlusion

Retinal vein occlusion (RVO) is a major cause of vision loss, primarily due to macular edema. The pathogenesis involves thrombus formation, leading to increased retinal capillary pressure and elevated vascular permeability. The first-line treatment for macular edema in RVO is intravitreal inhibition of vascular endothelial growth factor (VEGF), with aflibercept, bevacizumab, and ranibizumab being the most commonly used agents. In cases where VEGF inhibitors are insufficient, intraocular steroids serve as a second-line therapy to treat macular edema [147].

The TANZANITE phase II trial (NCT02303184) assessed suprachoroidal triamcinolone acetonide (SCTA) alongside IVT aflibercept injections versus aflibercept monotherapy for ME due to retinal vein occlusion (RVO) [148]; 84.1% of patients in the combination therapy group had resolution of ME compared to 50.7% of the monotherapy group (*p* = 0.001). Additionally, patients in the CLS-TA group required fewer IVT injections (*p* = 0.013) [148].

However, two subsequent phase III trials were terminated early because primary superiority endpoints were not met [125,149]. SAPPHIRE (NCT02980874) compared SC injections of TA with IVT aflibercept against aflibercept monotherapy. Preliminary data indicated that the combination treatment arm had a favorable safety profile, but without significant benefits over the monotherapy group [148]. TOPAZ (NCT03203447) assessed SC delivery of TA with IVT anti-VEGF (ranibizumab or bevacizumab) against a sham SC procedure with IVT anti-VEGF alone. TOPAZ was prematurely terminated due to the SAPPHIRE trial findings [149].

In contrast, Nawar and colleagues demonstrated that combining SCTA with IVT ranibizumab led to fewer overall injections and better visual acuity gains over 12 months compared to ranibizumab monotherapy [150]. Additionally, SCTA monotherapy studies have shown promising improvements in BCVA and CST [117,118]. However, further large-scale multi-center studies are needed to combine optimal combination therapies and the long-term safety of SCTA for RVO-associated ME.

### 8.2. Age-Related Macular Degeneration 

Age-related macular degeneration (AMD) is the leading cause of severe and irreversible vision loss worldwide [151]. This condition profoundly impacts the quality of life for affected individuals. AMD is classified into two types: dry (non-neovascular) and wet (neovascular). Dry AMD is characterized by the accumulation of drusen and the gradual thinning of the macula, leading to a slow progression of vision loss. The severe form of dry age-related macular degeneration (AMD), known as geographic atrophy, is characterized by extensive atrophy in and around the macula, which is crucial for central vision. This condition is irreversible, and no effective treatments are currently available to restore the vision [152].

In contrast, wet (neovascular) AMD involves the growth of abnormal blood vessels beneath the retina, which can leak fluid or blood, causing rapid and severe vision impairment. Advances in medical research have identified vascular endothelial growth factor (VEGF) as a crucial factor in the development of neovascular AMD. Inhibiting VEGF intraocularly has proven to be one of the most effective treatments available. The widespread adoption of anti-VEGF therapy has greatly improved the prognosis for patients with neovascular AMD, allowing the majority to maintain their visual function [153]. However, anti-VEGF therapy, administered via intravitreal (IVT) injections, has its drawbacks. The need for in-office procedures and patient compliance with frequent appointments every few weeks throughout the year pose significant challenges [154].

To address these limitations, SC delivery is a promising therapeutic avenue for neovascular AMD (nAMD). Current nAMD treatments utilize IVT delivery of anti-VEGF agents [153].

Axitinib is a tyrosine kinase inhibitor that acts with pan anti-VEGF activity. Preclinical animal studies demonstrated a favorable pharmacokinetic profile, with an almost 11-fold higher exposure of medication in the chorioretinal tissue with SC delivery compared to IVT [95,155]. OASIS was an open-label dose-escalation phase I/IIa trial (NCT04626128) that evaluated a proprietary axitinib suspension (CLS-AX) following anti-VEGF therapy (IVT Aflibercept) in patients with nAMD [156,157]. Data showed no serious adverse events and no dose-limiting toxicities, with a 77–85% reduction in treatment burden. Additionally, the 3-month extension study (NCT05131646) showed that 92% of participants did not require additional therapy at 3 months and 50% of participants at 6 months [119]. Mean BCVA and CST remained stable at 6 months. These positive results have led to ODYSSEY (NCT05891548), an ongoing phase IIb trial in which anti-VEGF-experienced participants with nAMD will receive either a combination of IVT aflibercept and SC axitinib or IVT aflibercept alone [120,156]. Recruitment has been completed, and the topline data is expected to be released in the third quarter of 2024.

Additionally, novel gene therapies such as the use of SC RGX-314 highlight new advancements that may reduce the frequency of injections [15]. For example, the ongoing AAVIATE (NCT04514653) trial will compare RGX-314 against monthly ranibizumab in patients with nAMD. SC delivery offers promising alternatives to IVT injections for nAMD, demonstrating the potential to reduce treatment burden and improve clinical sequelae.

### 8.3. Choroidal Melanoma 

Choroidal melanoma represents the most common primary intraocular malignancy in adults. In the presence of high suspicion for this malignancy, a biopsy is often recommended, utilizing techniques such as fine-needle aspiration or vitrectomy. The metastatic evaluation is critical due to the high incidence of metastatic disease, which occurs in approximately 25% of patients at five years and 34% at ten years post-diagnosis [158]. The liver is the primary site for metastasis, though other common sites include the lungs, bones, and skin. The evaluation also aims to rule out possible metastasis from other primary cancers to the choroid. Comprehensive evaluation includes physical examination, liver imaging (ultrasound, computed tomography, or magnetic resonance imaging), liver function tests, lung imaging (X-rays, computed tomography, positron emission tomography), and biopsy to confirm metastatic disease [159].

Treatment options for choroidal melanoma vary based on the size and location of the tumor as well as patient factors. Observation is reserved for benign choroidal tumors, small suspicious melanomas near the macula, or large melanomas in medically unstable patients. Radioactive plaque brachytherapy is the most common treatment, providing a good tumor control rate of approximately 90%, though it may limit vision in up to 50% of patients due to complications like optic neuropathy, maculopathy, radiation cataract, and neovascular glaucoma [160]. Charged-particle radiation offers a slightly higher tumor control rate of about 98% but carries risks of anterior segment damage, including neovascular glaucoma and chronic dry eye. Enucleation can be considered for large melanomas with non-useful vision. Pre-enucleation radiation does not improve mortality but decreases local orbital recurrence. External-beam radiotherapy is now less commonly used and less effective, with stereotactic radiotherapy and gamma knife radiosurgery being preferable alternatives. Transpupillary thermotherapy is employed for small choroidal melanomas to control recurrence at the tumor margin and as an adjuvant to brachytherapy, as it has a higher local tumor recurrence rate when used alone [161].

Beyond retinal pathologies, SC drug delivery has opened new possibilities in choroidal melanoma. While brachytherapy is the most common treatment for choroidal melanoma, it is invasive and associated with risks mentioned previously [160].

Belzupacap sarotalocan, also known as AU-011 or bel-sar, is being studied as a leading alternative to radiotherapy for choroidal melanoma. A recent rabbit model showed that bel-sar is well distributed in the choroid with histological tumor and cancer cell regression [162]. Additionally, Kang et al., demonstrated successful SC delivery of resin beads and fluorescent molecules to the site of intraocular melanomas using a microcatheter, with no inflammatory reactions [163].

Preliminary data from a phase 1/2 clinical trial (NCT03052127) of SC bel-sar with laser activation reported statistically significant reductions in tumor growth rate (-0.483 mm over 12 months, *p* = 0.018), 71% visual acuity preservation, and favorable safety profiles [123,124]. Of the 43 patients (56 total) that had juxta-foveal tumors, two patients had treatment-related serious AEs (vision loss). Similarly, interim results from another phase II trial (NCT04417530) confirmed the drug’s tolerability and lack of treatment-related serious AEs [164,165]. Given these promising results, a randomized masked phase III clinical trial (NCT06007690) is currently recruiting patients and comparing high- and low-dose bel-sar SC injections against a sham SC injections and laser [122]. Patient recruitment started as of May 2024.

The preliminary success of SC delivery of bel-sar offers a promising alternative to traditional brachytherapy for choroidal melanoma. Ongoing trials will further determine its efficacy and potential to become a new standard in ocular oncology.

### 8.4. Glaucoma

Topical anti-glaucoma medications exhibit low biological bioavailability that require daily administration, leading to poor compliance and potential local and systemic side effects [166]. For glaucoma treatments, delivery into the anterior SCS adjacent to the ciliary body is ideal, as SCS expansion is believed to improve aqueous humor outflow and/or reduce aqueous humor production [167,168]. Thus, the ideal glaucoma treatment likely requires a high-viscosity solution with low degradation and clearance to prevent posterior distribution.

**Figure 5:**
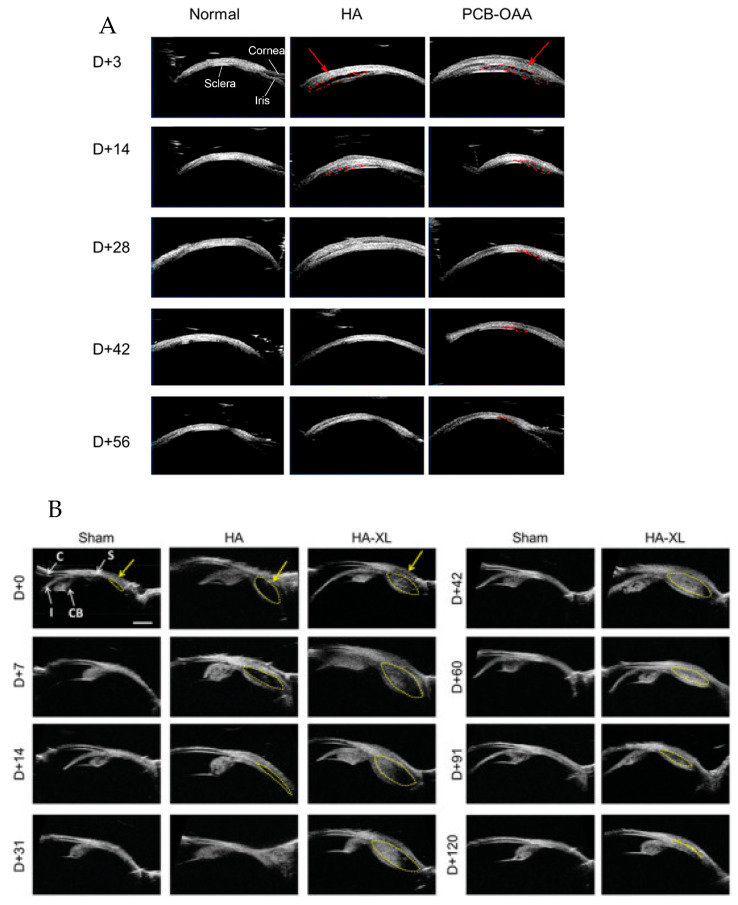
Ultrasound biomicroscope images of hydrogel-injected eyes. (**A**) The red arrow indicates the approximate location of the injection site and the red dashed line roughly outlines the enlarged suprachoroidal space after injection of the hydrogels [168]. Used under CC BY-NC-ND 4.0. (**B**) The yellow arrow indicates the approximate injection site, and the yellow dashed line roughly outlines the expanded suprachoroidal space [169]. Used under CC BY-NC-ND 4.0.

Pre-clinical animal studies have demonstrated promising results. Chae et al. demonstrated that a cross-linked in-situ-forming hyaluronic acid hydrogel injected into the SCS reduced IOP for up to 4 months [169]. This was supported by Chiang and colleagues, who showed that a SC delivery of brimonidine-loaded poly (lactic acid) microspheres lowered IOP for up to 1 week [170]. Both studies demonstrated favorable safety profiles with no major AEs. Mild AEs included mild conjunctival redness, minor hemorrhage, and injection site healing delays. Additionally, Hao et al. (2022) demonstrated that their polyzwitterion polycarboxybetaine hydrogel was well-tolerated and decreased IOP for up to 6 weeks, which was proportionally correlated with SCS expansion (Figure 5) [168].

Chiang and colleagues’ recent report on their monolithic hydrogel implant and custom-designed injector system for glaucoma demonstrates exciting potential for long-term IOP reduction [22]. A polyethylene glycol hydrogel was selected for its ease of manufacturing, biocompatibility, and permeability. Additionally, the bespoke 27-gauge microneedle of 0.8 mm is the first ever use of a microneedle to deliver a solid implant (as opposed to a liquid formulation) into the SCS. Chiang et al.’s microneedle utilizes an oblique needle angle to deliver the implant without kinking, as opposed to current FDA-approved microneedles, which are held perpendicular to the sclera. Further preclinical animal studies are needed to evaluate the implant and microinjector’s safety and therapeutic efficacy. These innovative gels present intriguing therapeutic potential in SC delivery, highlighting the potential for long-term delivery in a minimally invasive manner.

### 8.5. Regulatory Status of Suprachoroidal Technologies

The regulatory and approval statuses of suprachoroidal technologies reflect significant advancements and ongoing efforts to bring innovative treatments to market. Currently, the only FDA-approved suprachoroidal intervention is XIPERE (SC-TA) for the treatment of macular edema associated with uveitis based on the PEACHTREE trial [129]. XIPERE (or ARVN001) is currently undergoing non-US market testing in China and South Korea [171]. Additionally, Clearside Biomedical is also currently pursuing the use of XIPERE for diabetic macular edema in Australia, New Zealand, and Asia [172].

Several other investigational treatments are in various stages of clinical trials. Another promising candidate, OXU-001 (Dexamethasone), is currently in phase II trials for diabetic macular edema [111]. Belzupacap Sarotalocan (AU-011), a viral-like drug conjugate, is in phase III trials for treating choroidal melanoma, following promising results from the COMPASS trial [164]. RGX-314, an AAV-based gene therapy, is being explored in phase II trials for neovascular age-related macular degeneration (AMD) in the AAVIATE trial and for diabetic retinopathy in the ALTITUDE trial [69,71]. Similarly, CLS-AX (Axitinib), a tyrosine kinase inhibitor, is in phase II of the ODYSSEY trial, targeting neovascular AMD and showing potential to reduce treatment burdens [156].

**Figure 6:**
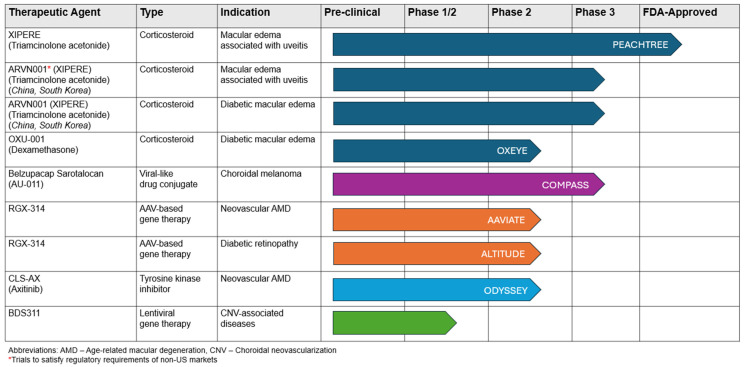
This figure illustrates the clinical development stages of various therapeutic agents delivered via suprachoroidal injection for different ocular conditions. The progress of these therapies demonstrates ongoing advancements and the potential for new treatments for ocular diseases.

Despite recent advancements, several barriers to market entry persist. Regulatory hurdles involve lengthy approval processes to ensure safety and efficacy, particularly for innovative treatments like gene therapies and nanotechnology. Additionally, the high cost of developing new treatments can limit accessibility, although there is ample research into alternative non-proprietary SC injectors. Furthermore, ensuring long-term safety and efficacy is crucial to be favored over intravitreal injections, the long-standing and widely accepted treatment modality for posterior ocular disease. For an overview of current clinical trials evaluating SC delivery, please see Figure 6.

### 8.6. Limitations of Clinical Applications

The use of SC injections should be based on clinical evidence and individual patient considerations. Patients with a history of ocular disease such as active ocular infections, uncontrolled systemic disease, or a history of ocular procedures such as pars plana vitrectomy surgery, scleral buckling, glaucoma shunts or plaque radiotherapy were excluded in many clinical trials [129,131,149]. Additionally, although a history of myopia or glaucoma was not an exclusion criterion for SCTA trials, patients with high myopia (≥ 6.00 diopter) or IOP > 22 mmHg were excluded [129,131]. More real-world efficacy studies in these populations are needed, and clinical judgement should be applied on a case-by-case basis.

Currently, clinical studies have demonstrated that using a 900 µm microneedle and injecting in the superior temporal quadrant is appropriate for most patients (71%, 6 trials, *n* = 581 injections) [33,109,173]. Retrospective analyses have found no correlations have been found between needle length and age, race, ocular disorder, refraction, visual acuity, IOP, retinal central subfield thickness, or lens status [109]. A post hoc analysis found that 71% of baseline SC injections used the 900 µm needle [21,173]. However, injection site can vary depending on the indication for SC injection. In SC injections of belzupacap sartalocan for choroidal melanoma, the ideal injection site is likely the two quadrants closest to the tumor location. To reduce the need for needle switching, the physician may first re-adjust their positioning to ensure true perpendicularity of the injector with the site of injection and that a dimple is present on the scleral surface. Overall, SC microneedles had low technical difficulties and high acceptance; when surveyed, 84% of physicians did not find SC injections more challenging than other ocular injection modalities [21]. Moving forward, specific criteria for quadrant selection or needle switching (i.e., the number of attempts with a 900 µm needle before moving to a second 1100 µm needle) is needed for patients when the SCS is not readily accessed in the first attempt.

## 9. Suprachoroidal Delivery Advancements: A 2023–2024 Overview

Since 2023, the field of suprachoroidal deliveries has shown significant advancements in treating ocular diseases. Diabetic macular edema has made the most progress within the last year, with numerous ongoing clinical trials on varying therapeutic agents, including SC aflibercept, dexamethasone, and fluocinolone acetonide implants [23,111,114,140]. Additionally, substantial growth in pre-clinical models highlights promising new drugs such as plasma kallikrein and integrin inhibitors [115,116]. Neovascular AMD has also shown ample progression with two currently ongoing phase II clinical trials and numerous gene therapy advancements [71,73,121,156]. For non-infectious uveitis related macular edema, post hoc analyses, and retrospective observational studies continue to support the efficacy of SCTA across varying clinical factors and real-world settings [105,106,107,109,114].

Recent advancements have addressed several key obstacles in the effectiveness of SC injections, improving their therapeutic potential. Further development of different SC delivery vessels makes this delivery method more accessible, as more devices, both proprietary and open source, are created. Additionally, novel sustained-release formulations including hydrogels and implantations will potentially reduce the frequency of injections required and treatment burden. Remaining challenges include long-term safety and efficacy, the standardization of procedures, and managing immune responses. While initial studies and preliminary results are promising, there needs to be continued comprehensive long-term data to monitor for potential delayed adverse events, ensure sustained therapeutic benefits and evaluate durability. Additionally, ensuring the reliability of SC injections across different patient populations and clinical settings will continue to be a technical challenge. Ongoing refinement of delivery devices and training protocols for healthcare providers in this novel treatment field will help minimize inconsistencies. Finally, managing immunogenicity remains a critical area for gene therapies. Further strategies to mitigate these responses may include utilizing fewer immunogenic vectors, lowering viral doses, and incorporating immunosuppressive agents. While significant advancements have been made, ongoing research and development is essential in order to further improve therapeutic benefits.

## 10. Conclusions

The suprachoroidal space is an up-and-coming ocular delivery route due to its proximity to chorioretinal tissues and favorable safety profile. Minimally invasive microneedle injections have been well-developed and FDA-approved for CLS-TA in patients with macular edema secondary to non-infectious uveitis. Numerous clinical trials are currently underway for other chorioretinal conditions such as diabetic macular edema, choroidal melanoma, and age-related macular degeneration. Additionally, pre-clinical studies have shown significant potential with various gene therapies, gel polymers, and implants. This review has underscored the rate at which suprachoroidal therapies have developed, many of which hold enormous potential in the treatment of vision-threatening conditions. In conclusion, SCS is a promising new drug delivery route that will become an integral part of chorioretinal therapies.

## Figures and Tables

**Table 1:** Comparison of different ocular administration methods.

Administration Method	Advantages	Disadvantages
Intravitreal Injection [36]	Office-based, outpatient procedure	Requires frequent injections
	High bioavailability (Bypasses corneal and blood–retinal barriers)	Limited drug penetration to the retina and choroid
	Widely used and well-established procedure	Potential for complications such as endophthalmitis, increased intraocular pressure, and cataracts
Subretinal Injection [37]	Direct delivery to target retinal cells, ensuring high drug concentration	Limited distribution of the injectate
	Reduced immunogenicity	Higher risk of complications such as retinal detachment and infection.
		Invasive surgical procedure that needs a hospital setting
Suprachoroidal Injection	Targeted delivery to the retina and choroid	Potential for injection site discomfort and transient IOP changes
	High bioavailability (Bypasses corneal and blood–retinal barriers)	Newer and less-established procedure
	Office-based, outpatient procedure	Requires specialized equipment

**Table 2:** Comparison of ocular gene therapy methods.

	Examples	Advantages	Disadvantages
	Adenovirus Adeno-associated virus Lentivirus	Tissue-specific tropisms	Potential immunogenic responses
Viral Vectors [15,37]		Improved specificity	Limited size
		Increased transduction	Risk of mutagenesis
			Expensive production
Non-Viral Vectors [27,62]	Naked DNA Nanoparticles (Protein, polymer, liposome-based)	Lower risks of toxicity and immunogenicity	Low specificity
		Larger sizes	Less stable than viral vectors
		Cheaper production	

**Table 3:** Suprachoroidal delivery advancements: A 2023–2024 overview.

Drug	Treatment	Key Findings	Study Design	Study
Macular Edema (ME) Secondary to Macular Edema (ME)				
TA	SCTA, 4 mg (0.1 mL of 40 mg/mL) at Day 0 and Week 12 vs. SC sham injection at Day 0 and Week 12 In patients with concurrent systemic corticosteroid or steroid-sparing therapy vs. no use	Patients with no steroid-sparing therapy, at Week 24: - ETDRS letter change: + 15.6 (SCTA) vs. + 4.9 (control) (*p* < 0.001). - CST change: -169.8 µm (SCTA) vs. - 10.3 µm (control) (*p* < 0.001). - Need for rescue therapy: 14.7% (SCTA) vs. 69.4% (control.) Among patients receiving steroid therapy, at Week 24: - ETDRS letter change: + 9.4 (SCTA) vs. - 3.2 (control) (*p* = 0.019). - CST change: -108.3 µm (SCTA) vs. - 43.5 µm (control) (*p* = 0.190). - Need for rescue therapy: 10.7% (SCTA) vs. 80% (control). - AE: no serious AEs in either group.	Post hoc analysis of phase III clinical trial (PEACHTREE)	Merrill, 2023 [105]
TA	SCTA, 4 mg (0.1 mL of 40 mg/mL) at Day 0 and Week 12 vs. SC sham injection at Day 0 and Week 12 In patients with different anatomic subtypes of uveitis	At Week 24: - BCVA: Similar gains (+ 12.1 to + 15.9 letters) across all anatomic subtypes (anterior, intermediate, posterior, and panuveitis). - CST: Change from baseline (− 120.1µm to − 189.0 µm) favored the treatment arm and achieved statistical significance for all subgroups except for Pts with anterior NIU. - Mean IOP: Similar increases (0.5 to + 3.1 mmHg) across all anatomic subtypes. - AE: Similar incidence among subtypes.	Post hoc analysis of phase III clinical trial (PEACHTREE)	Shah, 2024 [106]
TA	SCTA, 4 mg (0.1 mL of 40 mg/m) at Day 0 and Week 12 In patients with a CST ≥ 300 µm since 1/1/2022	At 36 d: - BCVA: Improved from 0.57 ± 0.48 to 0.47 ± 0.22 logMAR. - CST: Mean reduction in CME by 74.9% (range 32–100%). - IOP: Increased by 2.9 ± 3.9 mmHg; 1 Pt had sustained IOP that required therapy. - AEs: Minimal IOP elevation even in steroid-responsive patients overall. - Follow-Up: 36.0 ± 26.7 d after injection; 8 non-responders, 2 partial responders, 15 complete responders.	Observational, retrospective study	Zhou, 2023 [107]
TA	SCT, 4 mg (0.1 mL of 40 mg/m) at Day 0 and Week 12 vs. SC sham injection at Day 0 and Week 12 Patients with CST ≥ 300 µm and BCVA of ≥ 5 and ≤ 70 ETDRS who received ≥ 1 study treatment in either PEACHTREE or AZALEA	At 24 weeks: - BCVA improvement: 47.4% SCTA Pts (*n* = 95) gained ≥ 15 ETDRS letters from baseline vs. 16.7% of control group Pts (*n* = 60; *p* < 0.001) - +9.6 EDTRS letter gain in the SCTA group at Week 4 and + 13.9 letters at Week 24. - CST reduction: − 163.9 µm (SCTA) vs. − 19.3 µm (control) (*p* < 0.001). - AE: No treatment-related serious AEs. - Incidence of elevated IOP: 12.6% (SCTA) and 15.0% (control). - Incidence of cataract formation/worsening: 7.4% (SCTA) and 6.7% (control).	Post hoc analysis of 2 phase III trials (PEACHTREE and AZALEA)	Yeh, 2023 [108]
TA	SCTA, 4 mg using a hollow microneedle	- Looked at NIU, DME, and RVO. - 84% of physicians did not find the SC injections more challenging than other ocular injections. - Needle Length: 71% of baseline injections used the 900 μm needle, 29% used the 1100 μm needle. - Gender: 76% of female Pts used 900 μm needles compared to 66% for male patients (*p* = 0.006). - Quadrant: 78% of injections in the superotemporal quadrant used 900 μm needles compared to 65% in the inferotemporal quadrant (*p* < 0.001). - No correlation between needle lengths and BCVA, CST, and IOP. - AE: Lower incidences of IOP elevation, exacerbation of glaucoma, and cataract development compared to intravitreal or periocular corticosteroid injections.	Retrospective analysis of 6 clinical trials: AZALEA, PEACHTREE, TYBEE, TANZANITE, SAPPHIRE, and TOPAZ.	Wan, 2020 [109]
**Diabetic Macular Edema**				
TA + bevacizumab	SCTA, (0.1 mL; 40 mg/mL) + IVTB (1.25 mg of 0.05 mL) vs. SC sham injection of TA + IVTB (1.25 mg of 0.05 mL)	- BCVA change: −0.37 ± 0.24 letters (*p* < 0.001) (combination) vs. −0.20 ± 0.20 (monotherapy) (*p* = 0.004) at 12 weeks (between-group analysis (*p* = 0.014)). - Combination group showed improvements from baseline at Week 4 (*p* = 0.046) and 24 weeks later (*p* < 0.001). - Mean CST changes: Monotherapy: -76 μm (95% CI 15 to 138, *p* = 0.015) after 4 weeks and 108 μm (95% CI 51 to 164, *p* < 0.001) after twelve weeks. Combination: 337 ± 107 μm (*p* < 0.001) after 4 weeks and 348 ± 132 μm (*p* < 0.001) after completing the study. - Higher CST reduction in combination group vs. monotherapy group (*p* = 0.019). - AEs: No serious AEs related to treatment in either group.	Clinical trial (Phase II/III) (IRCT20200314046761N1)	Fazel, 2023 [110]
TA + aflibercept	SCTA, 2.4 mg (2.4 mg/60 µl) via microcatheter vs. SCTA, 4 mg (4.0 mg/100 µl) via microcatheter	- Ongoing study - 24-week randomized, two-arm, single-masked study to evaluate the safety and efficacy of two doses of SCTA administered through a novel proprietary microcatheter device in patients with previously treated DME.	Clinical trial (phase II) (NCT05512962)	Oxular Limited, 2024 [111]
TA + aflibercept	Single SC injection of 4 mg (100 µl) of TA using a proprietary Everads injector	- Ongoing study - 6-week, open-label study aiming to evaluate the safety and performance of the Everads injector.	Pilot device study (NCT06314217)	Everads Therapy, 2024 [23]
Aflibercept	SC injection Aflibercept (80 µg/eye; 2 µl injection) or sterile PBS (control) vs. IVT Aflibercept (80 µg/eye; 2 µl injection) vs. sterile PBS (control)	- SC injections were feasible in a mouse CNV model. - Significant suppression of leaky CNV lesions in both delivery methods. FA Leakage area reduction: - Day 3: IVT 58.6% (*p* < 0.01) vs. SC 49.8% (*p* < 0.05) - Day 7: IVT 54.2% (*p* < 0.05) vs. SC 76.8% (*p* = 0.014)	Preclinical animal study	Cerrada-Gimenez, 2023 [112]
Dexamethasone	SC injection of OXU-001 (dexamethasone formulation) vs. IVT dexamethasone implant	- Ongoing study - 52-week 2-part trial. Part A: Open-label randomized, single dose 2 treatment arm comparing 2 dose levels of SC OXU-011. Part B: Randomized, masked, active comparator, single dose, 3-treatment-arm study of 2 dose levels of OXU-001 and IVT dexamethasone implant. - Primary outcome: Treatment-emergent adverse event, mean change in BCVA, CST, time until follow-up rescue treatment needed.	Clinical trial (phase II) (NCT05697809)	Oxular Limited, 2024 [113]
Fluocinolone Acetonide (Iluvien®)	Single SC delivery of a 0.19 mg fluocinolone acetonide implant. (releases 0.25 µg/day)	- BCVA: Significant median increase of 3 lines; from pre-operative median BCVA 0.07 to post-operative BCVA of 0.15 (*p* = 0.02). - Median CMT: 25% reduction (544 to 404 microns, *p* = 0.4). - IOP: 50% of Pts had a transient rise in IOP (< 10 mmHg) that resolved within 3 weeks with anti-glaucoma drops. - AE: 2/10 phakic Pts developed nuclear sclerosis grade I. None of the 3 Pts with existing sclerosis showed progression through the end of the follow-up period.	Retrospective interventional non-comparative case series	El Rayes, 2023 [114]
CLS-301	Single bilateral SC injection of 100 µL of CLS-301 (4 mg/eye) in rabbit models at 16 weeks. (*n*=12 rabbits)	- Safety: Well-tolerated with no overt signs of toxicity or intraocular inflammation. - Drug distribution: Mean CLS-301 levels in the central retina were maintained 30- to 550-fold higher than the in-vitro IC50 values (0.5–5 ng/mL for blocking cell adhesion to vitronectin) for up to 4 months. - At day 112, mean drug levels in the central and peripheral retina were 1–2 orders of magnitude higher than IVT values (674 ng/gm and 955 ng/gm, respectively). - Sustained and high drug levels were observed in the RPE/choroid/scleral area and retina. - Compartmentalization: Minimal systemic and anterior segment exposure with low and sporadic CLS-301 levels were detected in the plasma, vitreous humor, and aqueous humor.	Preclinical animal study	Kansara, 2023 [115]
BCX- 4161	Single bilateral SC injection of 100 µL of BCX4161 (0.5 mg/eye) in rabbit models up to 12 weeks.	- Safety: Well-tolerated with no overt signs of toxicity. - Distribution: Sustained and high exposure of BCX4161 was observed in the chorioretinal area. - The retina had 1 to 2 orders of magnitude higher mean concentration than the vitreous humor. - Low levels of BCX 4161 were observed in aqueous humour and plasma samples. - C_max_: Peripheral retina: 75 µg/gm at 24 hrs post-dose, Central retina: 13 µg/gm at 24 hrs post-dose. - Concentration at Day 84: Retinal concentration levels were 1–2 orders of magnitude higher than the in-vitro IC99 (100 nM) levels. Central retina: 30 µg/gm, Peripheral retina: 21 µg/gm.	Preclinical Animal experimental study	Muya, 2021 [116]
**Macular Edema due to Retinal Vein Occlusion**				
TA	SCTA, 4 mg (0.1 mL of 40 mg/mL)	- Mean BCVA gain: 68.7% participants had a >15 letter gain at Week 1, 62.5% at Month 1, 50% at Months 2 and 3. - >70 ETDRS letter score: 81.25% at Week 1, 75% at Months 1, 2, and 3. - Mean CST reduction: Associated with improvements in BCVA. - Mean IOP: No significant changes, with an increase ranging from 0.75 mmHg at Week 1 (*p* = 0.09) and 0.5 mmHg at 3 months (*p* = 0.72).	Clinical trial (Phase I/II)	Ali, 2023 [117]
TA	SCTA, 4 mg (0.1 mL of 40 mg/mL)	- Mean BCVA gain: significant improvement from baseline at 3 months (*p* = 0.003) - Mean central retinal thickness reduction: significantly decreased from 342.2 ± 40.2 µm to 289 ± 47.5 µm at 3 months (*p* = 0.002).	Single-center, case series	Muslim, 2022 [118]
**Neovascular Age-related Macular Degeneration**				
Axitinib	Subjects who received SC 0.1 mg, 0.5 mg, and 1.0 mg Axitinib in the parent study will be followed for an additional 12 weeks	- AEs: No serious AEs, no treatment emergent AEs related to study treatment, no dose limiting toxicities, and no adverse events related to inflammation, vasculitis or vascular occlusion. - 90% reduction in treatment burden from the average monthly injections before CLS-AX - Injection-free rate (Cohorts 3 and 4): 88% up to Month 5 and 75% up to Month 6. - Mean BCVA and CST remained stable at the 6-month mark	Observational, prospective cohort of a phase II clinical trial (OASIS) (NCT05131646)	Clearside Biomedical, 2024 [119]
Axitinib	SC injection of 1.0 mg axitinib with a flexible dosing regiment Vs. IVT injection of aflibercept Q8W	- Study Ongoing - Randomized, double-masked, parallel-group, active-controlled study - Outcomes: BVCA, CNV lesion size on fundus fluorescein angiography, number of study drug injections and supplemental therapies, serious AEs and treatment-emergent AEs.	Clinical trial (phase II) ODYSSEY (NCT05891548)	Clearside Biomedical, 2024 [120]
RGX-314	Cohort 1: SC 2.5 × 10^11^ RGX gc/eye Vs. Cohort 2: SC 5 × 10^11^ RGX gc/eye Vs. Cohorts 1 and 2 control: Monthly 0.5 mg SC Ranibizumab Cohort 3: SC 5 × 10^11^ RGX gc/eye vs. SC 5 × 10^11^ RGX gc/eye in Pts with positive neutralizing antibody	- Pending full results - As of Nov 2021, RGX-314 was well tolerated in 50 patients dosed in Cohorts 1–3. - 4 serious non-treatment-related adverse events were reported in 4 patients. - For Cohorts 1 and 2, all common treatment emergent AEs were mild at 6 months through the study. - 23% (7/20) patients had mild intraocular inflammation, which all resolved with topical corticosteroids. - Stable BCVA and CRT at 6 months in RGX314-dosed patients. - 29% (4/14) of Cohort 1 and 40% (6/15) of Cohort 2 patients received no further anti-VEGF injections after RGX-314 administration at 6 months.	Clinical trial (phase II) AAVIATE (NCT04514653)	AbbVie, 2023 [69] Khanani, 2022 [70]
AAV-v128, AAV8 vector	Single SC injection of 100 μL of AAV8 or AAVv128 (3.5 × 10^13^ vg/mL) (*n* = 2 monkeys) Single SC injection of 100 μL of AAV8 or AAVv128 (1 × 10^12^ vg/mL) (*n* = 4 rabbits)	- CNV lesions: Absence of Grade IV lesions in the AAVv128-treated group at 35-days and 49-days. AAV8 group had 31.25% and 41.67% of Grade IV lesions at 35- and 49-days respectively. - Transduction: AAVv128 had a 4-fold increase in photoreceptors, RPE, and horizontal cells compared to AAV8 in mice. - eGFP fluorescence: Significantly increased with a smaller AAVv128 vector dose (3.5 × 10^12^ vg/eye) compared to AAV8 (7 × 10^12^ vg/eye). - Anti-VEGF: Protein levels in the aqueous humor were higher AAVv128-treated NHPs compared to AAV8-treated NHPs.	Preclinical animal study	Luo, 2024 [73]
Poly (β-amino ester) nanoparticles	SC injections of 50 µL (19.2 μg), 100 µL (38.4 µg), and 200 μL (76.8 µg) of pCAG-GFP-Z1 *n* = 25 human-sized minipig eyes	- GFP expression: Widespread in photoreceptors and RPE cells after a single SC injection. Increasing the volume of the injectate did not significantly improve the uniformity of GFP expression. Multiple injections at different locations reduced variability in GFP expression. - 2 weeks after a single SC injection, GFP expression was detected in all parts of the retina with considerable within-eye and between-eye variability. - 12 weeks after injection, GFP levels were comparable to those at two weeks. - Tolerance: No signs of retinal toxicity. Histological analysis showed normal retinal appearance with no inflammatory cells.	Preclinical animal study	Shen, 2024 [121]
BD311	Single SC injection of integration deficient lentiviral vector expressing VEGFA antibody, 500 uL	- Looking at choroidal neovascularization associated diseases such as nAMD, DME, and RVO. - Recruiting participants, results pending study completion.	Clinical trial (Phase I)	Shanghai BDGene, 2022 [78]
**Choroidal Melanoma**				
AU-011	SC high-dose belzupacap sarotalocan (bel-sar) treatment and laser vs. SC low-dose bel-sar treatment and laser vs. Sham injection and sham laser	- Currently recruiting patients as of May 2024, target enrollment = 100. - Outcomes: tumor progression, visual acuity at 52 weeks.	Clinical trial (phase III) (NCT06007690)	Aura Biosciences, 2024 [122]
AU-011	Up to 2 cycles of 1 of 3 dose levels and repeat dose regimens of SC injections of AU-011 treatment with 2 laser applications	- Statistically significant reduction in the tumor growth rate (−0.483 mm/yr, *p* = 0.018) - Visual acuity preservation rate of 71%. - Favorable safety and tolerability profile with most AEs being transient and without clinical sequelae. - 2 patients had treatment-related serious AEs of vision loss (baseline 43/56 patients had juxta-foveal tumors)	Clinical trial (phase I/II) (NCT03052127)	Aura Biosciences, 2021 [123,124]
AU-011	3 cycles of 3 weekly SC AU-011 injections (max. dose 80 µg) and 2 laser applications vs. Plaque radiotherapy	- Terminated due to low enrollment. - This trial was based on patients who had received Belzupacap Sarotalocan in a previous clinical trial from the same industry sponsor. - The study intended to assess visual acuity at 5 years of time.	Case-Control (NCT05266430)	Aura Biosciences, 2023 [125]
**Miscellaneous**				
SC implant for Glaucoma	SC spacer implant made of photo-crosslinked polyethylene glycol (PEG) delivered via a custom-designed microneedle.	- Successful delivery and placement of PEG hydrogel implant within the SCS - Distribution: The implants of different lengths (15, 30, 37.5, and 45 mm) showed proportional increases in SCS thickness, cross-sectional area, and volume occupied. - No adverse events reported		Chiang, 2024 [22]
AAV8 vector	Comparison of 3 different doses of SC RGX-314 (AAV8), one of which will be infused with topical steroid	- Investigating diabetic retinopathy - Recruitment ongoing Preliminary results (3 months): - Diabetic retinopathy severity: 33% improvement in treatment arm (≥2 improvement in diabetic retinopathy severity score) vs. 0% in in control arm. - AEs: no intraocular inflammation, commonly observed AEs were not considered treatment-related.	Clinical trial (Phase II) ALTITUDE (NCT04567550)	Abbvie, 2023 [71]
Microneedle device trial	50-100 µl of sodium fluorescein (0.1%), indocyanine green (0.0074%), or 1.0 µm fluorescent (645 nm) polystyrene microspheres	- 0.2 × 0.9 mm needles with a sharp tip beveled at 12.5o was used. - A 25-gauge microvitrectomy cannula (5 mm long) was slid over the 34-gauge needle. The metal end of the microvitrectomy cannula was adjusted to expose either 700 or 900 µm of the 34-gauge needle tip exposed. - Epoxy resin connected the valved end of the cannula to the hub of the 34-gauge needle. - The SC needles were used to inject NaF, indocyanine green or fluorescent polystyrene microspheres into 40 rabbits, and 40 monkeys, and enucleated pig and human eyes. - Only 2 or 3 occasions where the SC needle needed to be slightly repositioned - No instances of inadvertent IVT injection.	Preclinical animal study	Katz, 2023 [24]

Abbreviations: SC—suprachoroidal, IVT—intravitreal, Pt—patient, TA—triamcinolone acetonide, AAV—adenovirus-associated virus, SCTA—suprachoroidal triamcinolone acetonide, NIU—non-infectious uveitis, ME—macular edema, DME—diabetic macular edema, RVO—retinal vein occlusion, AE—adverse events, IOP—intraocular pressure, BCVA—best corrected visual acuity, CST—central subfield thickness.

## Data Availability

Not applicable.
